# Determination of Reference Values of the Masseter Muscle Stiffness in Healthy Adults Using Shear Wave Elastography

**DOI:** 10.3390/ijerph18179371

**Published:** 2021-09-05

**Authors:** Anna Olchowy, Mieszko Więckiewicz, Andrzej Malysa, Cyprian Olchowy

**Affiliations:** 1 Department of Experimental Dentistry, Wroclaw Medical University, 50-367 Wroclaw, Poland; ania.olchowy@gmail.com (A.O.); m.wieckiewicz@onet.pl (M.W.); andrzejmalysa@o2.pl (A.M.); 2 Department of Oral Surgery, Wroclaw Medical University, 50-367 Wroclaw, Poland

**Keywords:** masseter muscle, masticatory system, normal values, reference values, shear wave elastography

## Abstract

Shear wave elastography (SWE) is an objective and reliable method for the assessment of muscles and internal organs. Every organ exhibits its own stiffness characteristics and hence requires individual reference values. We aimed to determine the reference values of stiffness of the masseter muscle in healthy adult individuals using SWE. We analyzed the data of 140 participants (74 men, 66 women) with a median age of 50 years. The overall mean elasticity was 10.67 ± 1.77 kPa. The average values were lower by 2.25 kPa (9.15%) in women compared to men (9.48 ± 1.47 kPa vs. 11.73 ± 1.27 kPa; *p* < 0.0001). The values of stiffness increased with age, with a correlation coefficient of about 0.35 and a *p* < 0.0001. Age was a significant influencing factor of masseter muscle stiffness. The left and right masseters had similar stiffness. We conclude that stiffness values are significantly lower in women than in men with a difference of 9%. Age significantly influences the stiffness of masseter muscle, and the values of stiffness increase significantly with age, particularly in men. However, further studies are required to determine the precise ranges of stiffness accounting for age and sex in healthy subjects and people with disorders and conditions of the masticatory system.

## 1. Introduction

Up to date, several ultrasound elastography techniques have been used for the evaluation of muscle stiffness. These include transient elastography, acoustic radiation force impulse elastography, real-time tissue elastography, and real-time shear wave elastography (SWE). Although SWE has been recently introduced to clinical practice, it has been validated and is gaining attention among practitioners. An SWE device produces mechanical vibration sources which radiate low-frequency shear waves inside tissues, creating two intense plane shear waves [1]. These waves propagate through soft tissues, promoting their distortion adequately to their degree of stiffness. Then, the waves are registered by an ultrafast scanner. SWE allows determining the actual elastic modulus of tissues and recording the stiffness (in kPa) of the region of interest (ROI) in an organ. The technique is reproducible, operator independent, and quantitative [2]. An SWE device is integrated into an ultrasound system with standard ultrasound probes. Hence, SWE can be carried out as part of an additional routine examination or during a standard examination.

Clinical applications of SWE primarily include the evaluation of muscles and internal organs. However, every organ exhibits its own stiffness characteristics and therefore requires individual reference values. The reference values for muscles have been mainly established for healthy people. Ewertsen et al. [3] determined the reference values for neck and shoulder muscles; Wang et al. [4] attempted to determine the reference ranges of stiffness for the upper trapezius during different degrees of shoulder abduction; Lallemant-Dudek et al. [5] aimed to define the reference values for healthy muscles and muscles affected by spastic cerebral palsy. Their study focused on the long head of the biceps brachii and medial gastrocnemius. Regarding internal organs, the values for the liver and breasts have been well established for both healthy people and people presenting pathologic conditions [6,7]. In many diseases (e.g., breast cancer), SWE helps to make a diagnosis, determining the stage of the disease, and evaluating the response to treatment [8].

Taking into account the characteristics of SWE, we decided to use it for the assessment of the masticatory muscles. Patients with temporomandibular disorders (TMD) often suffer from hypertrophy and increased tension of the masseter muscle [9,10]; however, hypertrophy is not always associated with TMD and its etiology remains unexplained. These symptoms can be monitored by SWE, and the response to treatment can be evaluated [11]. Furthermore, SWE can be used to assess the condition of the masseter muscle by a trained dentist during check-up visits [12]. For this reason, we attempted to establish the reference values for a normal masseter muscle tissue in healthy adult individuals, which can help to differentiate any disorders and conditions, using SWE. Additionally, we investigated the reference values in terms of age and gender, which are factors affecting the stiffness of the masseter muscle.

## 2. Materials and Methods

We enrolled a total of 140 healthy adult volunteers (164 volunteered for this study and 140 were included based on the inclusion criteria). The percentage of excluded subjects was lower than in the general population [13] because we aimed to recruit only healthy people. Inclusion criteria for the study were as follows: age of 18 years or older, absence of any signs and symptoms suggestive of TMD according to the Diagnostic Criteria for Temporomandibular Disorders [14] protocol, and no previous diagnosis of TMD or treatment for this condition. People with neuromuscular disorders and/or rheumatoid disease, cancer, or inflammation in the facial region, pregnant and breastfeeding women, and those using muscle relaxants and/or other drugs that can alter the functioning of muscles were excluded. In addition, individuals with any abnormalities within the masticatory muscles, such as pain within the masseter and parafunctional oral habits, were not included. All participants took part in the study voluntarily and gave informed consent. The study was conducted according to the Declaration of Helsinki and was approved by the Bioethical Committee at Wroclaw Medical University (KB–592/2018). 

This study is a part of the research project titled, “Shear Wave Sonoelastography in the Diagnosis and Management of the Masseter Muscles Disorders”, funded by the National Science Centre, Poland (funding ID: PREL.B160.18.007).

**Figure 1:**
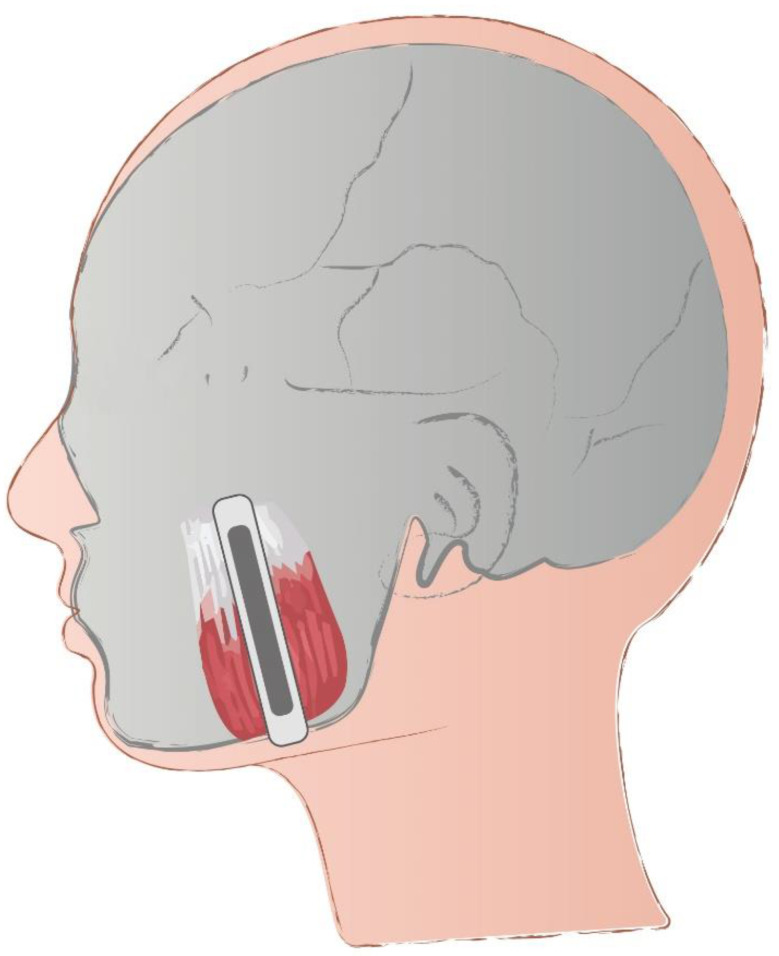
Placement of the probe on the masseter muscle during shear wave elastography examination.

During the study, each participant was first interviewed by a trained dentist and examined for the fulfillment of the inclusion criteria. If qualified for the study, the participant was referred for the SWE examination. All the SWE assessments were conducted by a single trained radiologist with seven years of experience in the technique. For each participant, three measurements each were taken in the right and left masseter muscle and averaged. In the previous pilot phase (unpublished data), measurements were made with a transducer placed longitudinally and transversely to the masseter muscle; however, no significant differences were found between measurements. Based on our experience, we recommend a longitudinal (parallel) placement as shown in Figure 1. In our opinion, such placement is more intuitive. Additionally, it is easier to achieve a 0° angle than a 90° angle of the probe in relation to muscle fibers. A similar approach was used by Chang et al. for measurement of the middle part of the masseter muscle [15]. While taking the measurement, the probe was placed parallel to the longitudinal axis of the masseter muscle in the widest part (the midpoint level) of the muscle in the belly. The middle part of the masseter muscle was identified while the patient clenched his/her teeth on the most protruding part of the muscle. 

A circular, 4 mm ROI was positioned in the center of the muscle tissue. The ROI of 4 mm was chosen to reflect the size of the masseter, avoiding the deep and superficial fascia of the muscles. It was located in an area of relatively uniform elasticity as guided by an SWE image and standard deviation (SD) of less than 30% of the mean elasticity value. During the SWE examination, the patients were asked to lie in a supine position, remain relaxed and comfortable, and refrain from swallowing. Before the examination, the probe was covered with an ultrasound gel to reduce the passage of air between the probe and the skin, which enabled good visualization. The tissues were not compressed during the examination.

The stiffness of the masseter muscle was measured with the Aixplorer Ultimate device (SuperSonic Imagine, Aix-en-Provence, France) using a high-frequency linear probe SL 18-5 (5–18 MHz). The obtained measurements were validated using the elasticity QA Phantom model 049A (Computerized Imaging Reference Systems, Inc, Norfolk, VA, USA).

Data were statistically analyzed using MedCalc v. 19.5.3 (MedCalc Software Ltd., Ostend, Belgium). Means and SDs were calculated. The Shapiro–Wilk test was used to analyze the data distribution. The hypothesis of normal distribution was rejected for all variables. The stiffness values were compared using the Mann–Whitney U test. Correlations between age and stiffness were checked with Spearman’s rank correlation coefficient. Regression models were built for predicting the stiffness value of the masseter based on age. A probability value lower than 0.05 was considered statistically significant.

## 3. Results

**Figure 2:**
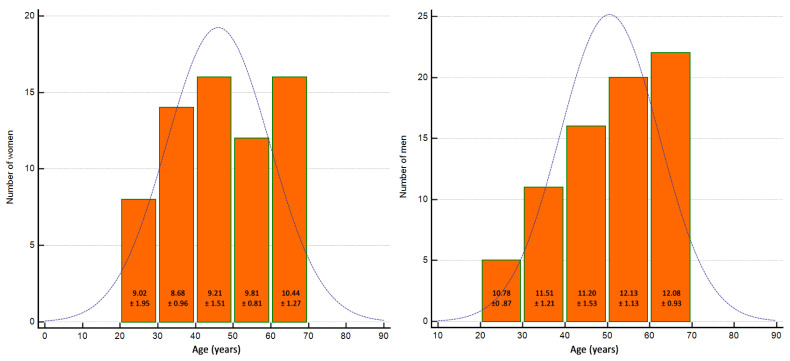
Age distribution in the study group by gender with a stiffness means expressed in kPa for age range.

**Figure 3:**
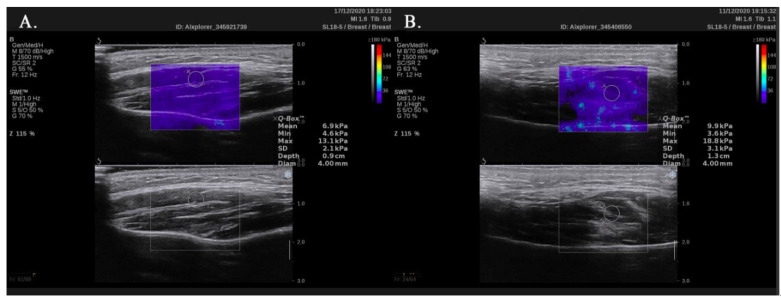
Shear wave elastography images of the masseter muscle of healthy volunteers: a 22-year-old woman (**A**) and 28-year-old man (**B**).

We analyzed data obtained from 140 healthy volunteers (74 men and 66 women) with a median age of 50 years (95% confidence interval (CI): 45.9–55). For women, the median age was 45 years (95% CI: 40–53) with a range from 22 to 65 years. Men were older with a median age of 54 years (95% CI: 47–57.9) and a range from 25 to 65 years. The age distribution is shown in Figure 2. The mean elasticity of all measurements was 10.67 ± 1.77 kPa. The values of stiffness by sex and side of the body are presented in Table 1. Figure 3 shows typical SWE images.

We investigated the relationship between age and stiffness values of the masseter muscle. Taking into account the side of the body, a moderate correlation was found between age and the stiffness value of the left masseter muscle (*r* = 0.353, 95% CI: 0.199–0.490, *p* < 0.0001). Similarly, a moderate correlation was found between age and the stiffness value of the right masseter muscle (*r* = 0.346, 95% CI: 0.192–0.485, *p* < 0.0001). As the comparison between left and right masseter muscle did not show any significant difference, we analyzed the stiffness values of left and right masseter together to calculate the correlation coefficients by sex. In women, a moderate correlation was found between age and the stiffness of the masseter muscle (*r* = 0.449, 95% CI: 0.232–0.623, *p* = 0.0002). On the other hand, a weak correlation was found in the case of men (*r* = 0.265, 95% CI: 0.0384–0.465, *p* = 0.0227).

Regression models were built for predicting the stiffness value of the masseter based on age separately for men and women. In each model, the stiffness values were set as a dependent variable (Y), and age as an independent variable (X). For women, the regression equation was as follows: *y* = 7.3351 + 0.04663 × *x*. The average stiffness value regardless of age was 7.33 kPa, which increased by 0.05 kPa for every additional year. Age significantly influenced the stiffness values (*p* < 0.0001). The unadjusted *R*-squared was calculated as 0.1870, and the model thus explained approximately 18% of the variability. For men, the regression equation was as follows: *y* = 10.2745 + 0.02881 × *x*. The average stiffness value regardless of age was 10.27 kPa, which increased by 0.03 kPa for every additional year. Similar to women, age significantly influenced the masseter muscle stiffness in men (*p* < 0.0001). The unadjusted *R*-squared was 0.0709, and the model thus explained approximately 7% of the variability.

## 4. Discussion

The reference values of the masseter muscle stiffness have not been established so far in the literature. Our study showed that the mean stiffness value in men was 11.73 ± 1.27 kPa, and in women was 9.48 ± 1.47 kPa. Age and sex had a significant impact on muscle stiffness. The average stiffness values were lower by 2.25 kPa (9.15%) in women compared to men. The values increased with age with a correlation coefficient of about 0.35 and a *p*-value below 0.0001. Thus, age was identified as a significant influencing factor of masseter muscle stiffness.

SWE is gaining importance in oral and facial imaging. Despite the insufficient evidence for its use in determining the stiffness of masticatory muscle, SWE has been widely applied over the past few years. However, there is still a need for large studies to establish the reference values of stiffness. For this reason, we conducted this study on 140 healthy adult individuals using SWE. Few reports providing stiffness values can be found in the literature [16]. Nevertheless, some factors can affect the results, which include the method used, equipment, age, sex, concomitant diseases, medications, and the degree of muscle tension. In Table 2, we summarize the results of previous research on masticatory muscle stiffness in healthy subjects with measurements taken using the Aixplorer device. It can be noted that the mean stiffness values range from 10.0 to 11.46 kPa. We used the same device in the present study and also expressed the results in kPa for better comparison with previous results, given that stiffness measured with different devices can produce different values [16]. In the Aixplorer device, SWE is performed using SuperSonic shear imaging. This means that acoustic radiation force induces displacement in the examined tissues and generates perpendicularly propagating shear waves. Next, those shear waves are detected by longitudinal ultrasonography waves that propagate faster than shear waves. The Aixplorer device provides quantitative real-time mapping of elastic modulus across soft tissue and objective stiffness value for the ROI expressed in kPa [1]. This method is characterized by low dependence on the operator, high repeatability, and the provision of quantitative assessment; however, a training and learning curve is required to use SWE.

Reports from the literature regarding the relationship between the stiffness of the masseter muscle and age are inconclusive. Some researchers report a positive correlation [19,20] and some report a negative one [21,22], whereas some claim that the stiffness values do not depend on age [2,23]. Our study revealed a significant correlation between the stiffness of the masseter muscle and age, with a correlation coefficient of 0.265–0.449. Thus, it can be interpreted that the stiffness of the masseter muscle increases with age.

The effect of age on stiffness has been reported in the literature for other muscles but not for the masseter muscle. Ekby et al. [18] examined the stiffness of biceps brachii in a group of 133 healthy subjects and reported similar findings to our study. They found that shear modulus values increased with age in full extension of the muscle, especially in people over 60 years of age. A possible explanation for increased stiffness at an older age is sarcopenia—a condition characterized by the loss of muscle mass and strength which is associated with increased collagen content in muscles [20,24]

On the other hand, Alfuraih et al. [21] reported contrasting results. They measured the stiffness of the quadriceps and biceps brachii in 77 healthy participants who were divided into three cohorts: young (20–35 years), middle aged (40–55 years), and elderly (above 75 years). Their results show a gradual decrease in resting muscle stiffness with a significant decline in the oldest group—the stiffness was 16.5% lower in the elderly participants compared to the young participants. They also revealed that sex and body mass index did not have any effect on muscle stiffness. Yoshida et al. [23] reported that the stiffness of gastrocnemius muscle was 9% lower in participants under 30 years of age than in those aged 30 years and older with a *p*-value of < 0.001, but they also found that the correlation between age and stiffness was not significant (*r* = 0.173 for men and *r* = 0.018 for women).

In the present study, the stiffness values were found to be higher in men, and therefore it is worth comparing the masseter muscle stiffness of men and women. For the masseter muscle, Arda et al. [2] reported that the stiffness was 4.6% lower in women compared to men. However, this difference was insignificant (*p* = 0.3). In addition, the correlation between age and masseter muscle stiffness was weak (*p* = 0.50).

Yoshida et al. [23] reported that the stiffness of the gastrocnemius muscle was 6% lower in women compared to men, with a *p*-value of 0.032. However, they did not examine the correlation as they did for age, which may suggest that this relationship might be not linear or could be confounded by outliers. Hence, it is difficult to draw conclusions about the sex-related differences in stiffness.

The present study has some limitations to be addressed. Firstly, SWE measurements were taken by only one experienced radiologist on a limited number of subjects. A question regarding the reliability of SWE might arise. However, SWE has already proven to be a reliable and accurate method for measuring the hardness/elasticity of soft tissues. It is also widely used in other therapeutic areas. Reports in the literature indicate that the measurements obtained using this technique are reliable and repeatable [11]. The results expressed in kPa to represent the hardness of tissues allow for comparisons between studies.

Olchowy et al. assessed the reliability of SWE measurements for the masseter muscle using intraclass correlation coefficients and observed excellent results [12]. Other researchers also proved this method to be reliable for different muscles [25,26,27]. Another limitation is that we examined only healthy adult subjects. The literature data show that the stiffness of the masseter muscle can increase in some disorders such as TMD [15] and after exercise [18], or decrease after applying therapeutic methods such as massage [11]. Therefore, this study shall be regarded as a reference for analyzing the disorders and conditions of the masseter muscle.

## 5. Conclusions

The present study showed that the stiffness of the masseter muscle was about 9% lower in women compared to men (9.48 ± 1.47 kPa vs. 11.73 ± 1.27 kPa; *p* < 0.0001). The masseter muscle on the left and the right side of the body had similar stiffness. Age significantly influenced the stiffness of the masseter muscle, and the value of this parameter increased significantly with age, particularly in men. However, further studies with larger samples are required to determine the precise ranges of stiffness accounting for age and sex in healthy subjects and people with disorders and conditions of the masticatory system.

## Figures and Tables

**Table 1:** Values of stiffness in the study group.

	Mean ± SD, kPa	Difference, kPa (%)	*p*-Value
Men (*n* = 148)	11.73 ± 1.27	2.25 (9.15%)	<0.0001
Women (*n* = 132)	9.48 ± 1.47		
Left total (*n* = 140)	10.66 ± 1.76	0.01 (0.09%)	0.9706
Right total (*n* = 140)	10.67 ± 1.78		
Women aged < Me	8.8 ± 1.4	1.36 (13.4%)	0.0001
Women aged > Me	10.16 ± 1.25		
Men aged < Me	11.43 ± 1.44	0.6 (4.9%)	0.0661
Men aged > Me	12.03 ± 1.01		

Abbreviations: Me, median; *n*, number of patietns, SD, standard deviation.

**Table 2:** Values of masseter muscle (expressed in kPa) stiffness from previous studies measured with the Aixplorer device.

Author	Quantity	Elasticity Values of the Masseter Muscle, kPa
Arda, 2011 [2]	127 healthy adult volunteers	Total: 10.4 ± 3.7 (range, 2–23) Men: 10.8 ± 3.9 (range, 4–20) Women: 10.3 ± 3.6 (range, 2–23)
Herman, 2017 [17]	176 healthy adult volunteers	10.0 ± 4.3, median 9.85
Olchowy, 2020 [11]	20 healthy adult volunteers	11.46 ± 1.55
Olchowy, 2021 [12]	51 healthy adult volunteers	Left: from 10.72 ± 2.32 to 10.67 ± 2.23 Right: from 10.88 ± 2.34 to 10.54 ± 2.38
Olchowy, 2021 [18]	40 healthy adult volunteers	Total: median 11.35 (interquartile range, 9.7–12.65) Left: 10.99 ± 2.04 Right: 11.01 ± 2.21

## Data Availability

The data presented in this study are openly available in FigShare at DOI: 10.6084/m9.figshare.16570467.
